# BFL1 modulates apoptosis at the membrane level through a bifunctional and multimodal mechanism showing key differences with BCLXL

**DOI:** 10.1038/s41418-018-0258-5

**Published:** 2018-12-18

**Authors:** Hector Flores-Romero, Olatz Landeta, Begoña Ugarte-Uribe, Katia Cosentino, Miguel García-Porras, Ana J. García-Sáez, Gorka Basañez

**Affiliations:** 1 Instituto Biofisika (CSIC, UPV/EHU), Parque Científico de la UPV/EHU, Barrio Sarriena s/n, Leioa, 48940 Bizkaia Spain; 2 Interfaculty Institute of Biochemistry, Eberhard Karls University Tübingen, Hoppe-Seyler-Str. 4, Tübingen, 72076 Germany; 3 Departmento de Bioquímica y Biología Molecular, Universidad del País Vasco (UPV/EHU), Barrio Sarriena s/n, Leioa, 48940 Bizkaia Spain

**Keywords:** Mechanism of action, Cancer, Membrane lipids

## Abstract

BFL1 is a relatively understudied member of the BCL2 protein family which has been implicated in the pathogenesis and chemoresistance of a variety of human cancers, including hematological malignancies and solid tumours. BFL1 is generally considered to have an antiapoptotic function, although its precise mode of action remains unclear. By quantitatively analyzing BFL1 action in synthetic membrane models and in cells, we found that BFL1 inhibits apoptosis through three distinct mechanisms which are similar but not identical to those of BCLXL, the paradigmatic antiapoptotic BCL2 family protein. Strikingly, alterations in lipid composition during apoptosis activate a prodeath function of BFL1 that is based on noncanonical oligomerization of the protein and breaching of the permeability barrier of the outer mitochondrial membrane (OMM). This lipid-triggered prodeath function of BFL1 is absent in BCLXL and also differs from that of the apoptotic effector BAX, which sets it apart from other BCL2 family members. Our findings support a new model in which BFL1 modulates apoptosis through a bifunctional and multimodal mode of action that is distinctly regulated by OMM lipids compared to BCLXL.

## Introduction

Apoptotic programmed cell death plays an essential role in many human physiological processes and pathologies [1]. Mitochondrial outer membrane permeabilization (MOMP) is an integral step in apoptosis which is regulated by BCL2 family proteins [2–4]. Members of this protein family are usually divided in three main groups based on their principal function and the presence of up to four BCL2 Homology motifs (BH 1–4 motifs) defined by sequence conservation:[2, 5] (i) BCL2-type repressors, which contain all four BH motifs and prevent MOMP (BCL2, BCLXL, BFL1 and others); (ii) BAX-type effectors, which contain BH1–3 motifs and directly elicit MOMP once they are activated (BAX, BAK and perhaps BOK); and (iii) BH3-only activators (BID, BIM, and others), which instigate the function of BAX-type effectors. In addition, most BCL2 family members contain a C-terminal Tail-Anchoring (TA) motif that is generally less well conserved at the sequence level, and typically addresses these proteins to the outer mitochondrial membrane (OMM) [5, 6].

Despite the relevance of the above classification, important questions remain regarding the molecular mechanisms by which BCL2 family proteins exert their biological function. One outstanding issue is to elucidate the exact pattern of interactions comprising the BCL2 interactome, particularly at the level of the OMM [4, 7]. From a structural viewpoint, all multidomain BCL2 family proteins display a hydrophobic groove centered on the BH1 motif that serves as a receptor-site for binding BH3 motifs of ligand partners [2]. In addition to this canonical BH3:groove binding interface, other noncanonical interaction surfaces have been identified in a variety of BCL2 family proteins. Two prominent examples are the BH4 and TA motifs of BCL2 and BCLXL, which can mediate heteromeric interactions with BAX implicated in apoptosis repression [8–10]. However, whether such noncanonical binding interfaces universally contribute to apoptosis inhibition by BCL2-type proteins remains unknown. Another often overlooked property of BCL2-type proteins is their ability to acquire proapoptotic phenotypes under certain circumstances [11–15]. Nevertheless, the fundamental mechanisms by which BCL2-type proteins shift from inhibiting to promoting apoptosis remain poorly understood.

BFL1 is one of the least extensively studied BCL2-type proteins [16, 17]. It is accepted that BFL1 prevents apoptosis under specific physiological settings, and that BFL1 contributes to tumour progression and drug resistance in selected types of human cancer [16–23], akin to BCL2, BCLXL and MCL1 [24]. However, the precise mode of action of BFL1 is less well understood than those of BCL2, BCLXL and MCL1. Here, using both minimalist and cellular systems, we identified common and distinguishing mechanistic traits between BFL1 and BCLXL in the attainment of their antiapoptotic function. We also report that the apoptosis-related lipids cardiolipin (CL) and oxidized CL (CLox) unleash a prodeath function in BFL1, but not in BCLXL, that is based in a noncanonical membrane-permeabilizing activity of the protein.

## Results

### BFL1 retrotranslocates nonactivated BAX from the membrane through a mechanism showing similarities and key differences with BCLXL

**Fig. 1:**
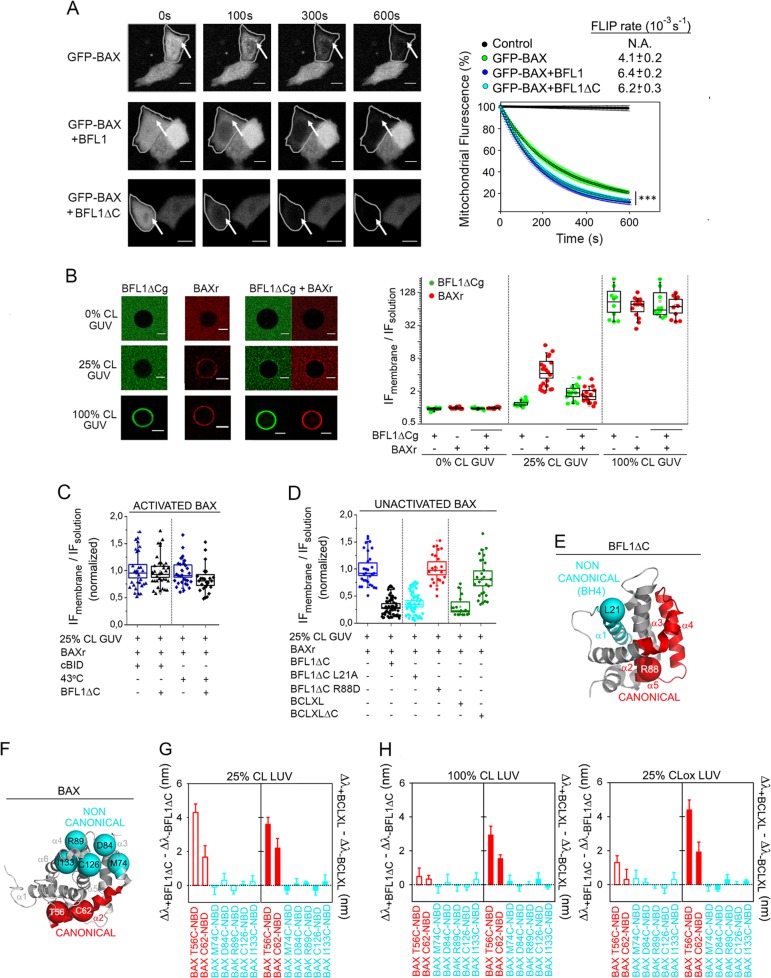
BAX retrotranslocation by BFL1. **a** FLIP assessment of GFP-BAX retrotranslocation from mitochondria to cytosol. Left: Representative images showing time-dependent FLIP of mitochondrial GFP-BAX in the absence (top) or presence of overexpressed BFL1 (middle), or BFL1ΔC (bottom). Arrows denote mitochondria. Scale bars, 10 µm. Right: Average FLIP kinetics for multiple cells (*n* > 20) expressing GFP-BAX either alone, or together with BFL1, or together with BFL1ΔC. Control represents GFP-BAX fluorescence in a neighbour unbleached cell (black line). Data correspond to three independent experiments and are expressed as mean ± S.D. ***p = < 0.001. **b** BFL1∆Cg and/or BAXr were coincubated with GUV containing different CL amounts at 37 °C, and protein binding to the GUV was determined by confocal fluorescence microscopy. Left: Representative GUV images. Scale bar 10 µm. Right*:* Ratio of maximum normalized integrated intensity values at the membrane (IF membrane) and in solution (IF solution). In this Box Chart representation, each dot represents individual (raw) data; the box represents the 96% confidence interval; inside the box, the media and median are represented by the small square and the line, respectively; and the errors correspond to 80% of the data. All data correspond to at least three independent experiments, with more than 10 GUV analyzed for each condition. **c**, **d** BAXr was incubated with 25%CL GUV in the presence or absence of BAX activators for 2 h at 37 °C (or at 43 °C in heat-activated BAX experiments), followed by further incubation with antiapoptotic proteins for another 2 h, and determination of the degree of BAXr binding to the GUV by confocal microscopy. **e** Structural representation of BFL1ΔC (PDB:2VM6) in grey, depicting L21 (cyan) and R88 (red) residues at putative noncanonical (cyan) and canonical (red) interaction surfaces of BFL1ΔC, respectively. **f** Structural representation of BAX (PDB:1F16) in grey, depicting residues localized at putative noncanonical and canonical interaction surfaces of BAX used to generate NBD-labelled monocysteine BAX variants for fluorescence mapping studies. **g** Fluorescence mapping analysis of BAX retrotranslocation from 25%CL LUV. Canonical (red) and noncanonical (cyan) NBD-labelled BAX monocysteine variants were incubated first with 25%CL LUV for 2 h at 37 °C, followed by further incubation with BFL1ΔC or BCLXL for another 2 h, and subsequent determination of NBD *λ*_max_ by fluorescence spectroscopy. **h** Fluorescence mapping analysis of BAX retrotranslocation from apoptotic-like LUV. Other details as explained in (**g**). In (**g**) and (**h**), *n* ≥ 3 technical replicates, and error bars, SE

BCLXL has been shown to keep BAX inactive in healthy cells via continuous retrotranslocation of BAX from the mitochondria into the cytosol [25]. Thus, we first analyzed whether BFL1 shares with BCLXL this antiapoptotic mode of action. GFP-BAX was ectopically expressed in BAX/BAK DKO HCT116 cells in the presence or absence of BFL1 under nonapoptotic conditions, and GFP-BAX retrotranslocation was quantitatively analyzed using Fluorescence Loss In Photobleaching (FLIP) (Fig. S1a, b). BFL1 accelerated the FLIP rate of mitochondrial GFP-BAX without increasing the FLIP rate of cytosolic GFP-BAX, akin to BCLXL [25] (Fig. 1a and Fig. S1c). Also similar to BCLXL, GFP-BFL1 itself showed a decrease in mitochondrial fluorescence by FLIP following a first-order kinetic, and BAX overexpression accelerated the FLIP rate of mitochondrial GFP-BFL1 (Fig. S1d). Next, we observed that BFL1 did not increase the FLIP rate of the GFP-BAX 1–2/L-6 mutant unable to establish canonical BH3:groove interactions due to intramolecular tethering (Fig. S1e) [25]. Since noncanonical interactions mediated by BCLXL TA motif (helix α9) contribute to BCLXL-mediated BAX retrotranslocation [26], we then examined the behaviour of a BFL1 variant lacking helix α9 (hereinafter named BFL1ΔC). Remarkably, BFL1ΔC accelerated the FLIP rate of mitochondrial GFP-BAX in a manner indistinguishable from the full-length protein (Fig. 1a).

**Fig. 2:**
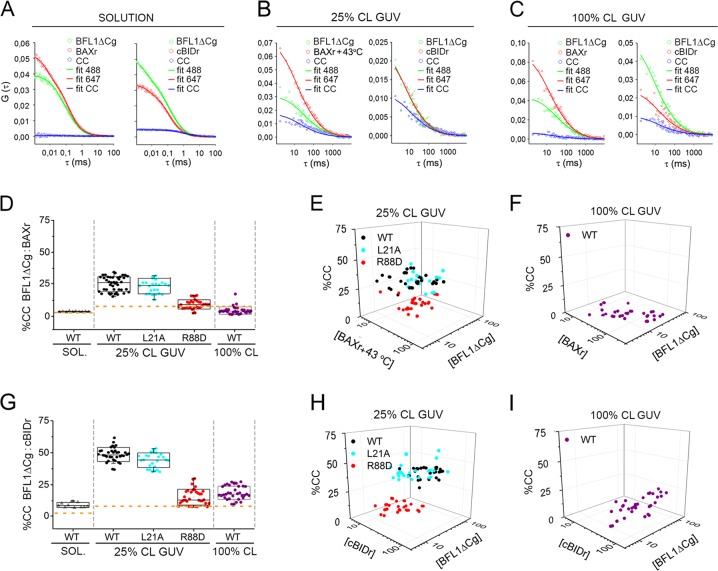
Assessment of BFL1∆C heterocomplex formation. **a**, **b**, **c** FCCS analysis of BFL1∆Cg:BAXr and BFL1∆Cg:cBIDr heterocomplexes in solution, in 25%CL GUV, and in 100%CL GUV. Dots represent raw data; straight lines, fitted auto-correlation curves (green and red), and cross-correlation curves (blue). **d**, **g** Quantification of BFL1ΔCg heterocomplexes. Box chart representations as described in Fig. 1b. Dashed orange lines indicate the minimum %CC considering heterodimer formation and the degree of labeling. **e, f, h, i** Heterocomplex formation in individual GUV represented on a 3D plot as a function of protein concentrations (molecules/µm^2^). All data correspond to at least three independent experiments, with more than 20 GUV analyzed for each condition. Error bars, SD

We next attempted to reproduce the BFL1-mediated BAX retrotranslocation process in a minimalist model system. Here, we incubated Giant Unilamellar Vesicles (GUV) containing different amounts of the mitochondrion-signature lipid CL with fluorescently-labelled and functional BFL1ΔCg and BAXr variants (Fig. S2a, b, c). Neither BFL1ΔCg nor BAXr bound significantly to OMM-like GUV lacking CL, but both of them detectably interacted with OMM-like GUV containing CL levels typically found in mitochondrial contact sites under healthy conditions (25%CL GUV, Fig. 1b) [27]. Importantly, coincubation with BFL1ΔCg reduced BAXr binding to such healthy-like GUV by ~ 65%. On the other hand, both proteins bound avidly to 100%CL GUV emulating CL microdomains found in mitochondria of apoptotic cells [28], and coincubation with BFL1ΔCg did not change BAXr binding to such apoptotic-like lipid membranes (Fig. 1b). Of note, BAXr alone extensively permeabilized 100%CL large unilamellar vesicles (LUV) (Fig. S2d) suggesting that activated BAX cannot be retrotranslocated by BFL1ΔC. To further examine this possibility, we examined the effect of BFL1ΔC on BAXr pre-incubated with 25%CL GUV in the absence or presence of cBID or mild heat (43 °C) (Fig. S2d) [2–4, 7]. BFL1ΔC did not reduce membrane binding of cBID- or heat-activated BAXr (Fig. 1c), but it did so for the case of nonactivated BAXr (Fig. 1d). Next, we generated two single-residue BFL1ΔC mutants: R88D, localized in the BH1 motif of the canonical BH3-binding groove (Fig. 1e, red);[2] and L21A, localized at the BH4 motif purportedly implicated in BAX retrotranslocation by BCL2 (Fig. 2e, cyan) [8]. Both mutants retained the global structure of the native protein (Fig. S2e). However, the noncanonical L21A mutant, but not the canonical R88D mutant, replicated the ability of BFL1ΔC to retrotranslocate nonactivated BAX from 25%CL GUV (Fig. 1d, cyan and red symbols, respectively). We also examined the behaviour of BCLXL and BCLXL lacking its TA motif (hereinafter termed BCLXLΔC). BCLXL, but not BCLXLΔC, efficiently retrotranslocated BAXr from 25%CL GUV (Fig. 1d, green symbols).

To further compare the BAX retrotranslocation processes mediated by BFL1ΔC and BCLXL, we performed BAX topology mapping studies. We used a recently validated set of NBD-labeled BAX monocysteine variants encompassing the canonical BAX BH3 motif (Fig. 1f, red) [29], and the noncanonical BAX surface that binds the BCL2 BH4 motif (Fig. 1f, cyan) [8]. Remarkably, addition of either BFL1ΔC or BCLXL to the set of BAX monocysteine mutants pre-incubated with 25%CL LUV increased the *λ*_max_ of the BAX T56-NBD variant and, to a lesser extent the BAX C62-NBD variant, both of which are localized at the canonical BAX BH3 motif (Fig. 1g, red bars, and Fig. S2g, red spectra). By contrast, BFL1ΔC and BCLXL did not induce significant λ_max_ changes in any noncanonical BAX variant examined (Fig. 1g, cyan bars, and S2g, cyan spectra). These data suggest that BFL1ΔC- and BCLXL-mediated BAX retrotranslocation is commonly linked to a local change in BAX conformation characterized by partial exposure of its BH3 motif. Next, we examined the behaviour of BCL2 proteins in LUV containing apoptotic-like lipid compositions. Since it is well known that CL becomes oxidized during apoptosis [30], and considering that it is technically feasible to oxidize CL incorporated in LUV in a controlled manner (unlike in GUV) [31], here we used 100%CL LUV and 25%CLox LUV. Interestingly, BCLXL, but not BFL1ΔC, increased the *λ*_max_ of BAX BH3 motif monocysteine variants with such apoptotic-like lipid membranes (Fig. 1h).

### BFL1ΔC forms canonical heterocomplexes showing preference for cBID over BAX which are stabilized in healthy-like lipid membranes

To obtain quantitative information on the ability of BFL1 to form stable complexes in aqueous and membrane environments we applied point and scanning Fluorescence Cross-Correlation Spectroscopy (FCCS), respectively. In aqueous solution, the BFL1ΔCg/BAXr pair produced flat CC curves (Fig. 2a, left) and a mean CC level close to background (Fig. 2d, grey symbols and Fig. S3a) indicating no detectable BFL1ΔC:BAX complex formation, which is as observed for BCLXL [32]. On the other hand, the BFL1ΔCg/cBIDr pair produced CC curves with small but significant positive amplitudes (Fig. 2a, right) and a modest mean CC level (Fig. 2g, grey symbols) indicating weak BFL1ΔC:cBID complex formation. In healthy like 25%CL GUV, BFL1ΔCg/BAXr and BFL1ΔCg/cBIDr pairs displayed CC curves with more positive amplitudes (Fig. 2b) and higher mean CC levels (Figs. 2d–h). Of note, as observed with BCLXL [31], mean CC levels were smaller for BFL1ΔCg combined with BAXr than with cBIDr (Figs. 2d–g, black symbols). Moreover, the noncanonical BFL1ΔCg L21A variant behaved as the native protein interacting with BAX (Fig. 2d, cyan symbols) and cBID (Fig. 2g, cyan symbols), whereas the canonical BFL1ΔCg R88D mutant totally lost heterocomplex-forming ability (Figs. 2d–g, red symbols). Next, we analyzed the behaviour of BFL1ΔC in apoptotic-like 100%CL GUV. Here, the BFL1ΔCg/BAXr pair produced flat CC curves (Fig. 2c, left) and a negligible mean CC level (Figs. 2d–f, purple symbols), whereas the BFL1ΔC/cBIDr pair produced CC curves with detectable positive amplitudes (Figs. 2c, right) and a small but significant mean CC level (Fig. 2g–i, purple symbols).

### BFL1ΔC self-associates into noncanonical homocomplexes in apoptotic-like lipid membranes

**Fig. 3:**
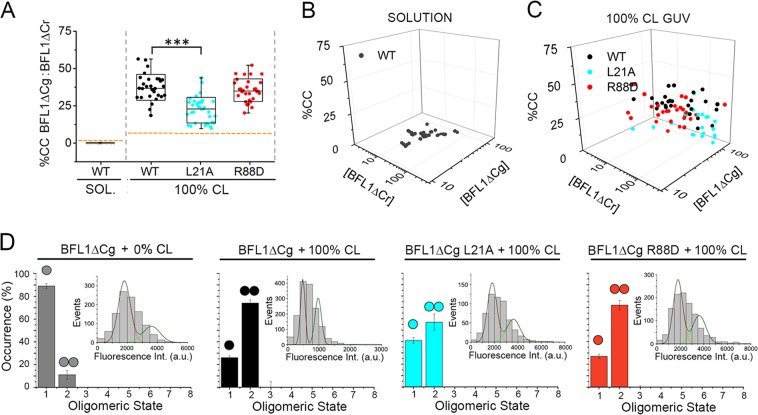
Assessment of BFL1∆C homocomplex formation. **a**, **b**, **c** FCCS analysis of BFL1∆Cg:BFL1∆Cr homocomplex formation in solution and in 100%CL GUV. Other experimental details as described in Fig. 2. **d** TIRF analysis of BFL1ΔC quaternary structure. Experiments with 0%CL SLB included 1% Ni-lipids to allow BFL1ΔC membrane binding. Monomers and dimers were calculated from the averaged distributions of species from different experiments, after correction for partial labeling. The error bars correspond to the average error for each species. Inset plots represent fluorescence intensity distribution of BFL1ΔCg or its variants particles (>500). The resulting histograms were fitted with a linear combination of two Gaussians to estimate the occurrence of particles containing one (orange) or two (green) labeled molecules. The area of the fitted Gaussians is proportional to the fraction of each species

We next addressed whether BFL1ΔC self-assembles into oligomers in different environments using FCCS. In aqueous solution, CC levels for the BFL1ΔCg/BFL1ΔCr pair were negligible at all concentrations examined (Figs. 3a, b, grey symbols). Interestingly, BFL1ΔC homocomplex formation was strongly promoted in apoptotic-like 100%CL GUV (Figs. 3a–c, black symbols). Moreover, the homocomplex-forming capacity of BFL1ΔC was totally retained by the R88D variant (Figs. 3a–c, red symbols), but not by the L21A variant (Figs. 3a–c, cyan symbols), suggesting BFL1ΔC forms homocomplexes in 100%CL LUV through noncanonical interactions. To investigate deeper the quaternary structure of BFL1ΔC, we used Total Internal Reflection Fluorescence (TIRF) with single-molecule sensitivity [33], and size-exclusion chromatography (SEC). TIRF experiments indicated that the majority of BFL1ΔC exists as a monomeric species in healthy-like membranes, whereas BFL1ΔC predominantly adopts a dimeric conformation in apoptotic-like membranes apparently via noncanonical interactions (Fig. 3d). SEC studies indicated that BFL1ΔC adopts a monomeric conformation in solution, whereas in the presence of 100%CL LUV the protein elutes as low-order oligomers (Fig. S4a). By contrast, BCLXL retained the same SEC elution profile with and without 100%CL LUV, whereas BAX shifted from being monomeric to forming high-order oligomeric structures (Fig. S4a). In further SEC experiments, the canonical BFL1ΔC R88D mutant behaved as the native protein further supporting that BFL1ΔC forms homocomplexes via noncanonical interactions, unlike the case of BAX (Fig. S4b) [2, 29].

### BFL1ΔC, but not BCLXL, forms pores in apoptotic-like lipid membranes showing distinguishing features with canonical BAX pores

**Fig. 4:**
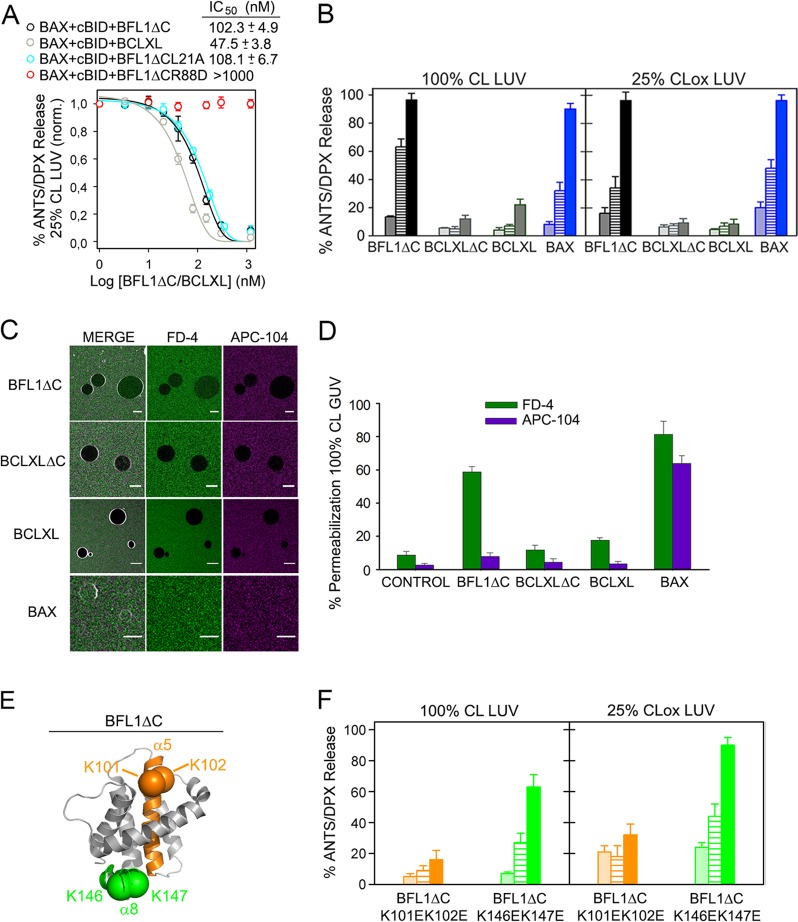
Effects of BFL1, BCLXL, and BAX on membrane permeability. **a** Inhibition of BAX-mediated membrane permeabilization elicited by BFL1∆C and BCLXL proteins in 25%CL LUV. *n* ≥ 3 technical replicates. Error bars, SE. **b** Membrane permeabilization elicited by BFL1ΔC, BCLXLΔC, BCLXL, and BAX in apoptotic-like LUV. Protein concentration: Dotted bars, 60 nM; Dashed bars, 120 nM; filled bars, 240 nM. *n* ≥ 3 technical replicates. Error bars, SE. **c** Representative images of 100%CL GUV treated with indicated BCL2 proteins in the presence of FD-4 (4 kDa) and APC-104 (104 kDa). Scale bars, 10 µm. **d** Percentage of 100%CL GUV with internalized FD-4 or APC-104. All data correspond to at least three independent experiments, with more than 25 GUV analyzed for each condition. Error bars, SD. **e** Structural representation of BFL1ΔC (PDB:2VM6) in grey, depicting two putative CL binding sites: K101K102 (green) and K146K147 (orange). **f** Membrane permeabilization elicited by BFL1∆C K101EK102E and BFL1∆C K146EK147E variants in apoptotic-like LUV. Other details as in (**b**)

Next, we examined the effects of BFL1ΔC, BCLXL, BCLXLΔC, and BAX on the permeability of healthy- and apoptotic-like lipid membranes. In healthy-like 25%CL LUV, BFL1ΔC inhibited the vesicular ANTS/DPX release elicited by cBID-activated BAX, albeit with a considerably smaller potency than BCLXL (Fig. 4a). The noncanonical L21A mutant, but not the canonical R88D mutant, fully reproduced this inhibitory capacity of BFL1ΔC (Fig. 4a). In apoptotic-like 100%CL LUV and 25%CLox LUV, BFL1ΔC and BAX by themselves efficiently released vesicular ANTS/DPX, while BCLXL and BCLXLΔC did not (Fig. 4b). The canonical BFL1ΔC R88D variant totally retained such autonomous membrane-permeabilizing activity, whereas the equivalent canonical BAX R109D mutant virtually lost all of it (Fig. S4c). Next, we assessed the internalization of two differently-sized fluorescent markers into 100%CL GUV: Fluorescent Dextran of 4 kDa (FD-4) and AlloPhycoCyanine of 104 kDa (APC-104). BFL1ΔC produced extensive internalization of FD-4, but not APC-104, indicating BFL1ΔC forms pores of relatively small size in 100%CL GUV (Figs. 4c, d). On the other hand, BCLXL and BCLXLΔC produced little internalization of both FD-4 and APC, while BAX elicited almost complete internalization of the two fluorescent markers (Figs. 4c, d) [34]. By inspecting the BFL1 sequence, we noted the presence of two consecutive lysine residues in BFL1 α5 helix (K101 and K102) and BFL1 α8 helix (K146 and K147), which may act as binding sites for dianionic CL, and which are absent in BCLXL and BAX (Fig. 4e). To evaluate the relevance of such potential CL binding sites on BFL1ΔC membrane activities, we generated BFL1ΔC K101EK102E and BFL1ΔC K146EK147E variants and examined their behaviour in liposome membrane permeability. The BFL1ΔC K101EK102E mutant lost virtually all capacity to permeabilize apoptotic-like LUV whereas the K146EK147E mutant retained most of this ability (Fig. 4f), while both mutants efficiently inhibited BAX permeabilizing activity in healthy-like LUV (Fig. S4d).

### Externalization and oxidation of mitochondrial CL unleashes a noncanonical proapoptotic function in BFL1, but not in BCLXL

**Fig. 5:**
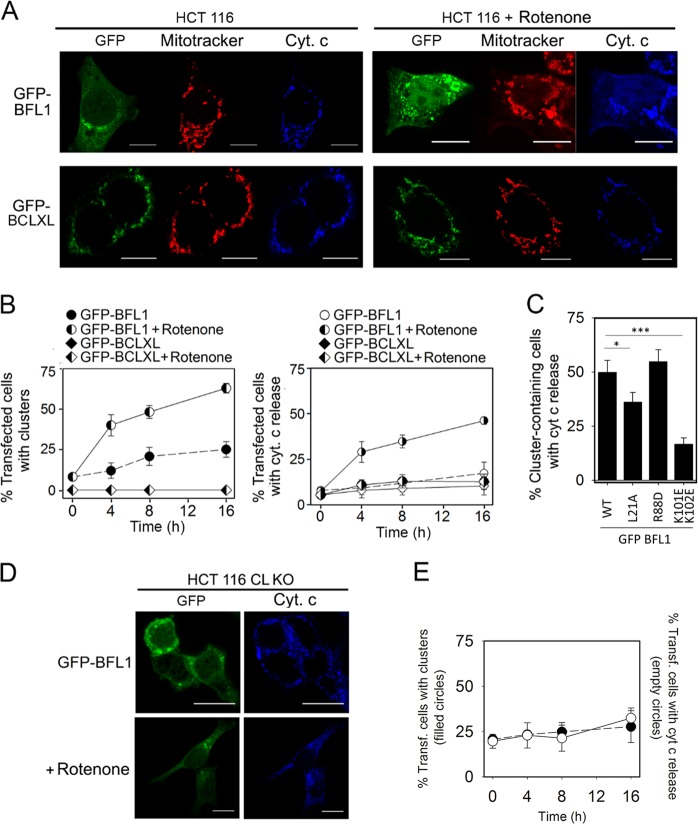
Effects of rotenone in HCT116 cells transfected with GFP-BFL1 or GFP-BCLXL. **a** Representative images of GFP-BFL1 and GFP-BCLXL intracellular distribution (green), cyt c localization (blue) and mitotracker intensity (red) in transfected HCT116 cells treated or untreated with rotenone (1 µM, 4 h). Scale bars, 10 µm. **b** Time-dependence of cluster formation and cyt c release in transfected HCT116 cells treated or untreated with rotenone (1 μM). **c** Quantification of the percentage of transfected cells presenting clusters and cyt c release after rotenone treatment (1 µM, 4 h). *p = < 0.05; ***p = < 0.001. **d** Representative images of GFP-BFL1 and cyt c intracellular distribution in HCT116 CL KO cells treated or untreated with rotenone (1 μM, 4 h). **e** Time-dependence of cluster formation (left) and cyt c release (right) in transfected HCT116 CL KO cells treated or untreated with rotenone (1 μM). In (**b**, **c**, **e**) at least 50 cells were analyzed per condition, and all the experiments were performed at least three times. Data are expressed as mean ± SD

It is well-known that rotenone induces mitochondrial CL oxidation and accumulation at the OMM [30, 35]. Thus, we decided to examine the impact of rotenone on the intracellular distribution of BFL1, BCLXL, and cyt c, in HCT116 cells ectopically expressing GFP-BFL1 or GFP-BCLXL. Remarkably, rotenone treatment caused GFP-BFL1 clustering and release of cyt c from mitochondria, two typical features observed with BAX during apoptosis (Figs. 5a, b and Fig. S5a). By contrast, rotenone did not trigger significant BCLXL clustering or cyt c release in cells expressing GFP-BCLXL, even after long-term treatment of these cells with the drug (Figs. 5a, b). Rotenone treatment did not produce clustering or cyt c release in HCT116 cells ectopically expressing GFP or Mito-GFP either (Fig. S5b, c). Interestingly, the relative capacities of GFP-BFL1 L21A, GFP-BFL1 R88D, and GFP-BFL1 K101EK102E mutants to form clusters and release cyt c upon rotenone treatment were in general agreement with the behaviour displayed by equivalent BFL1ΔC mutants in apoptotic-like lipid membranes (Fig. 5c and Fig. S5a–d). We also used HCT116 cells lacking CL (CL KO) to further test the specificity of the effects induced by rotenone on BFL1. Previous studies with such cells showed that CL deficiency does not reduce the apoptotic response or cBID-mediated BAX activation following treatment with TRAIL [36]. By contrast, lack of CL significantly decreases the capacity of BFL1 to form clusters and to release mitochondrial cyt c upon rotenone treatment (Figs. 5d–e).

**Fig. 6:**
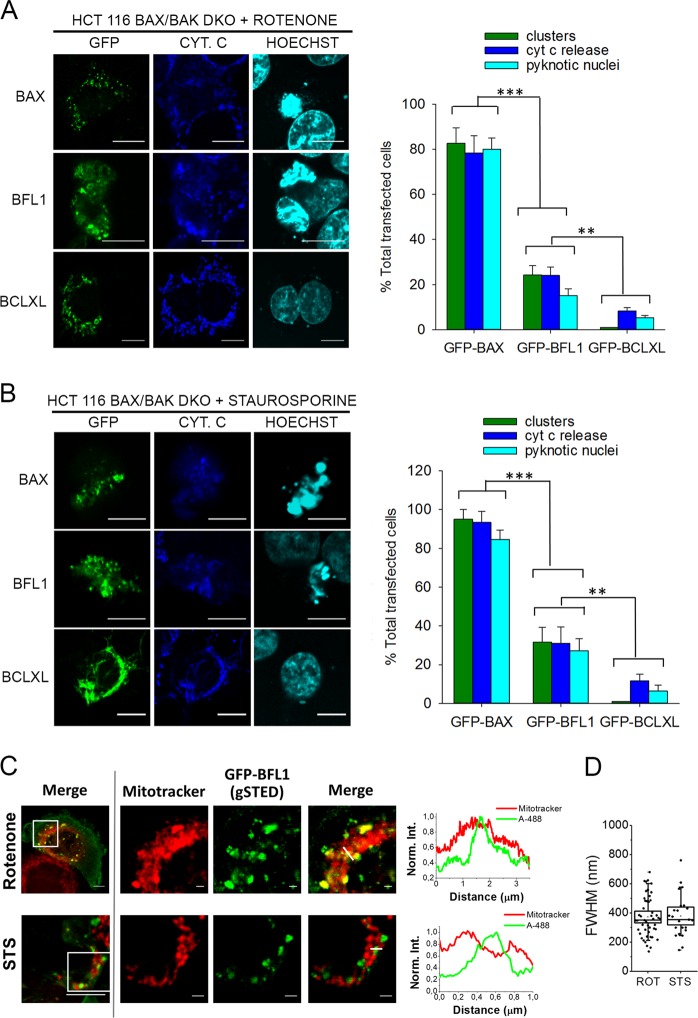
Effects of rotenone and staurosporine in HCT116 BAX/BAK DKO cells transfected with GFP-BFL1, GFP-BCLXL, or GFP-BAX. **a**, **b** Left, representative images of GFP-BAX, GFP-BFL1 and GFP-BCLXL intracellular distribution (green), cyt c localization (blue) and nuclear morphology (cyan) in transfected HCT116 BAX/BAK DKO cells treated with rotenone or staurosporine (1 µM, 6 h). Scale bars, 10 µm. Right, percentage of transfected HCT116 BAX/BAK DKO cells presenting clusters, cyt c release, or apoptotic nuclei after rotenone or staurosporine treatment (1 µM, 6 h). Data represent mean ± SD from three independent experiments (*n* > 50 on each repetition). **c** Left, representative merged images of rotenone- or staurosporine-treated HCT116 BAX/BAK DKO cells presenting GFP-BFL1 clusters. Scale bars, 5μm. The boxed regions of interest were zoomed out and recorded for confocal (mitotracker) and gSTED (GFP-BFL1-Alexa 488) observation. Scale bars, 1μm. Right, normalized intensity profiles of GFP-BFL1-Alexa 488 and Mitotracker along the pixels marked by the line on the zoomed merged images. **d** Spatial resolution (FWHM) of GFP-BFL1 clusters in the gSTED recorded images. Box chart representation as described in Fig. 1b

We next turned into HCT116 BAX/BAK DKO cells, and compared well-recognized apoptotic features in cells expressing GFP, GFP-BAX, GFP-BFL1 or GFP-BCLXL. As expected, rotenone treatment induced extensive BAX clustering, cyt c release, and pyknotic nuclei in HCT116 BAX/BAK DKO cells expressing GFP-BAX (Fig. 6a). Although less prominent than in the case of GFP-BAX, rotenone treatment also induced substantial BFL1 clustering, cyt c release, and pyknotic nuclei in HCT116 BAX/BAK DKO cells expressing GFP-BFL1, but not in those expressing GFP or GFP-BCLXL, or in the absence of rotenone treatment (Fig. S6b). Staurosporine has also been reported to produce mitochondrial CL externalization and oxidation, akin to rotenone [35]. In fact, we observed that staurosporine elicited very similar effects to rotenone in HCT116 BAX/BAK DKO cells expressing GFP-BAX, GFP-BFL1, or GFP-BCLXL (Fig. 6b and Fig. S6c). Finally, stimulated emission depletion (STED) nanoscopy revealed that BFL1 clusters are heterogeneous in shape and size and localize primarily at mitochondria (Figs. 6c, d).

## Discussion

BFL1 is an apoptosis-regulatory protein of the BCL2 family that has recently emerged as an important factor for oncogenesis and resistance to chemotherapy [16–23], akin to other BCL2-type proteins [24]. However, the mechanistic details of BFL1 action are much less well understood compared to those of BCL2, BCLXL, and MCL1. Here, using both minimalist and cellular systems, we revealed that BFL1 modulates cell viability at the OMM level through a bifunctional and multimodal mode of action presenting similarities but also key differences with BCLXL.

First, we showed that BFL1 shares with BCLXL the ability to transfer mitochondrial BAX back to the cytosol, an antiapoptotic mechanism of action that has been termed retrotranslocation or “Mode 0” [25, 37]. Moreover, as recently reported for BCLXL [32], our results with lipid-only model membranes provide evidence that BFL1 can directly retrotranslocate BAX. Interestingly, BCLXL requires its TA motif (helix α9) to retrotranslocate BAX, while BFL1 does not. Taking into account the similitude of BCLXL α9 with BAX α9 it has been proposed that BCLXL α9 plays a role in BAX retrotranslocation by competing with BAX α9 for binding to a common mitochondrial receptor or by directly extracting BAX away from the membrane [26, 38–40]. More recent evidence suggests that the driving force for BCLXL-mediated BAX retrotranslocation is the formation of high-affinity BCLXL homodimers in solution [32]. However, considering the unique nature of BFL1´s TA motif [41], it is perhaps not surprising that BFL1 does not share these features with BCLXL. We also demonstrated that BFL1ΔC retrotranslocates nonactivated, but not activated, BAX from OMM-like lipid membranes. This is in accordance with cellular studies showing that active BAX remains stationary at the OMM [25, 26, 38, 40]. Additionally, our fluorescence mapping studies revealed that the processes of BFL1- and BCLXL-mediated BAX retrotranslocation are communally linked to partial exposure of BAX BH3 motif. This is coherent with previous data showing that semi-exposure of BAX BH3 motif represents the very first step in the complex sequence of conformational changes through which BAX-type proteins acquire their active functional status [42]. Based in these observations, we hypothesize that under healthy conditions, OMM-associated BFL1/BCLXL engage in dynamic heterodimeric interactions with BAX at least in part due to incomplete exposure of BAX BH3 motif.

Next, we analyzed the capacity of BFL1 to form stable complexes with cBID (“Mode 1” antiapoptotic mechanism) or BAX (“Mode 2” antiapoptotic mechanism) [3, 43]. Quantitative FCCS studies showed that BFL1ΔC forms stable heterocomplexes preferentially with cBID over BAX in all environments examined. Our results are coherent with recent data obtained with BCLXL [32], but seemingly disagree with other studies [43, 44]. However, it is clear that unlike BCLXL [32], BFL1 does not require its α9 helix for stronger binding to cBID relative to BAX in a lipid membrane environment. Our FCCS results also speak against the notion that the BFL1 BH4 motif stabilizes the BFL1:BAX heterodimer at the membrane level, as proposed for BCLXL and BCL2 [8, 10]. On the contrary, our data support the view that BFL1 forms stable heterodimers at the OMM exclusively via the canonical BH3:groove interface. In addition, we showed that the apoptosis-related lipids CL and CLox obstruct stable BFL1ΔC heterodimerization and BFL1ΔC-mediated retrotranslocation of BAX. Since membrane lipid composition has little effect in BCLXL heterodimerization [32], it is tempting to speculate that accumulation of CL/CLox at the OMM during apoptosis hinders the capacity of BFL1, but not BCLXL, to heterodimerize with its proapoptotic ligands, and thereby blocks the antiapoptotic function of BFL1, but not that of BCLXL.

Last, we unveiled a novel lipid-triggered prodeath function of BFL1 linked to an intrinsic membrane-permeabilizing activity of the protein, which is absent in BCLXL, and differs from the canonical pore-forming activity of BAX. The differential effects displayed by BFL1ΔC, BCLXL, and BAX on the permeability of model membranes with healthy- and apoptotic-like lipid compositions are coherent with their dissimilar capacities to form heterocomplexes and homocomplexes in such distinct lipid membrane environments. However, characterizing BFL1ΔC activities in apoptotic-like lipid membranes we observed that BFL1ΔC forms homocomplexes and pores of smaller sizes than BAX and importantly, BFL1ΔC oligomerization does not rely in canonical BH3:groove interactions as in the case of BAX [2]. We also identified a potential CL binding site in BFL1 α5 helix comprising two consecutive lysine residues that plays a key role in BFL1´s membrane-permeabilizing function. Of note, these two lysine residues are not identified targets for BFL1 ubiquitination [14, 45, 46]. Accordingly, we showed that exposure and/or oxidation of mitochondrial CL by rotenone or staurosporine triggers noncanonical BFL1 clusterization and cyt c release which is not reproduced by a BFL1 variant lacking this putative CL binding site, or in cells devoid of CL. Despite BFL1 elicits these effects less potently than BAX, this issue could be related to BFL1 forming smaller pores than BAX in the OMM, and it is also compatible with the differential modulation of BAX and BFL1 by other cellular factors involved in the apoptotic pathway. Further studies are warranted to assess whether this lipid-triggered prodeath function is specific for BFL1 or can be extended to other BCL2 family proteins. Alternative mechanisms such as proteolytic cleavage may also unleash a pore-forming activity in BFL1 [14, 47], and perhaps even in BCLXL [48, 49], triggering their conversion into proapoptotic factors under specific circumstances.

**Fig. 7:**
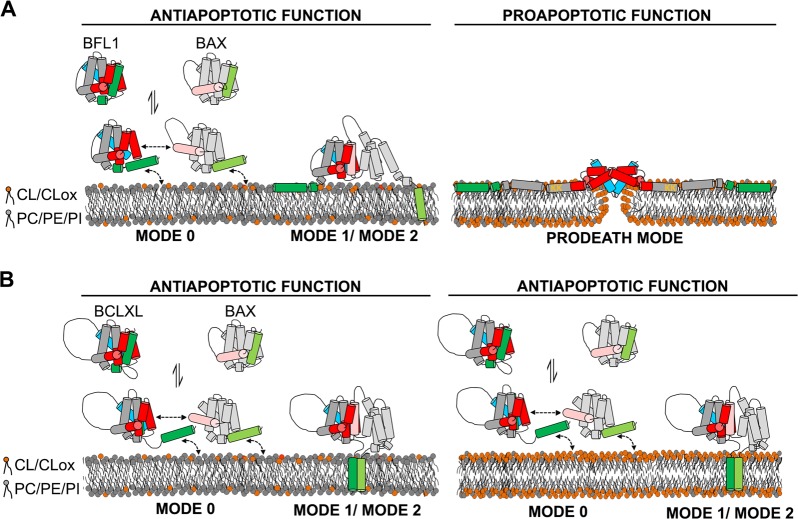
Proposed model for apoptosis-regulatory mechanisms of BFL1 and BCLXL depending on CL/CLox content at the OMM. **a** BFL1. Left: When the amount of CL/CLox at the OMM is low, BFL1 displays an antiapoptotic function through a trimodal mode of action consisting of (i) retrotranslocation of nonactivated BAX from the OMM to the cytosol (Mode 0), (ii) stable heterodimerization with cBID at the OMM (Mode 1, not depicted), and (iii) stable heterodimerization with activated BAX at the OMM (Mode 2). Right: Building-up of CL/CLox at the OMM unleashes a proapoptotic function in BFL1 through noncanonical BFL1 homo-oligomerization and interaction of BFL1 K101/K102 residues (orange) with CL/CLox leading to pore formation at the OMM. Of note, the precise conformation adopted by BFL1 at the OMM under these conditions is unknown, and thus several aspects of the structural model depicted here are speculative. **b** BCLXL. Left and Right: Independently of the amount of CL/CLox at the OMM, BCLXL displays an antiapoptotic function through a trimodal mode of action. In this schematic representation, BFL1/BCLXL/BAX are represented in grey, BFL1/BCLXL canonical grooves in red, BFL1/BCLXL BH4 motifs in blue, BFL1/BCLXL TA motifs in green, BFL1 K101/K102 residues in orange, BAX BH3 motif in light pink, and BAX TA motif in light green. Note that in the Mode 0 inhibitory mechanism BAX exposes its BH3 motif partially thereby only allowing transient heterodimerization with BFL1/BCLXL, while in the Mode 2 inhibitory mechanism BAX fully exposes its BH3 motif thereby allowing stable heterodimerization with BFL1/BCLXL. Note also that while heterodimerization between BFL1 and BAX/cBID exclusively relies in canonical BH3:groove interactions, heterodimerization between BCLXL and BAX/cBID may also rely in noncanonical interactions mediated by BCLXL´s TA motif as suggested in [32]

In summary, our findings support the following model (Fig. 7): (i) when the OMM contains relatively low levels of CL/CLox, BFL1 exerts its antiapoptotic function through three distinct modes of action, all of which are based in canonical BH3:groove heterodimeric interactions; and (ii) when the OMM accumulates CL/CLox, BFL1 unleashes a prodeath function through self-assembly of noncanonical BFL1 homocomplexes that breach the OMM permeability barrier. By contrast, increased CL/CLox levels at the OMM do not affect BCLXL function. We hypothesize that this bifunctional mode of action of BFL1 could be important to modulate the balance between anti- and proapoptotic BCL2 proteins under certain cellular stress conditions. We further speculate that under normal conditions the antiapoptotic function of BFL1 dominates over the proapoptotic one, thereby explaining why BFL1-defficient mice do not display evident signs of BFL1´s prodeath activity [50–52]. Nevertheless, as BFL1 overexpression is related with resistance to anticancer drugs [16–22], the potential therapeutic use of the lipid-mediated switch in BFL1 function reported here should not be dismissed.

## Materials and methods

### Chemicals and reagents

Egg phosphatidylcholine (PC), egg phospatidylethanolamine (PE), egg phosphatidylinositol (PI) and bovine heart cardiolipin (CL) were from Avanti Polar Lipids. MitoTracker Red CMXRos (Mitotracker), AlexaFluor 488 C_5_Maleimide (Alexa 488), Alexa Fluor 647 C_2_Maleimide (Alexa 647), N,N’-Dimethyl-N-(Iodoacetyl)-N’-(7-Nitrobenz-2-Oxa-1,3-Diazol-4-yl)Ethylenediamine (NBD), 1,1’-Dioctadecyl-3,3,3′,3′-Tetramethylindodicarbocyanine Perchlorate (DiD), 3,3′-Dioctadecyloxacarbocyanine Perchlorate (DIO), and Hoechst 33342 (Hoechst) were from Thermo Fisher Scientific. 1, 3, 6, aminonaphtalene-tri-sulphonate (ANTS) and p-xilene-bis-dipicolyinic acid (DPX) were from Molecular Probes. All other reagents were from Sigma-Aldrich.

### Retrotranslocation assays: FLIP measurements and plasmids

For FLIP experiments, HCT116 BAX/BAK DKO cells were seeded on 1 µm-slides in McCoy’s 5 A medium, grown for 20 h, transfected, and incubated for 6–16 h at 37 °C with 5% CO_2_. Confocal analysis was performed on a Zeiss LSM710 microscope equipped with a 63X oil objective and laserlines with excitation at 488, 561 or 633 nm. For GFP-BAX translocation studies, the cells were incubated with Mitotracker for 30 min prior to analysis. Then, the media was substituted for McCoy’s 5 A medium with 10 mM HEPES and without phenol red and incubated 30 min at 37°C with 5% CO_2_. Next, a single spot with a diameter of 1.3 µm within the nucleus was repeatedly bleached with two iterations of 100% power of a 488 nm laser line. The diameter of a single Region Of Interest (ROI) was 0.52 µm. Two images were collected after each bleach pulse, with 30 s between bleach pulses. After collecting 60 images, two separate measurements on the mitochondria were taken to analyze the fluorescence loss. Unbleached control cells were monitored for photobleaching due to image acquisition. FLIP rates were calculated from fluorescence intensity measurements using the Zeiss LSM software, setting the initial fluorescence to 100% signal. Plots are shown as normalized fluorescence over time. GFP-BFL1 plasmid was generated by PCR-based sequence amplification and posterior insertion in pEGFP-C3 (a generous gift from Richard Youle), whereas BAX and BFL1 were expressed using pCi-neo-myc vectors. GFP-BFL1ΔC (lacking the C-terminal 24 residues) was generated by PCR-based mutagenesis using the Quickchange mutagenesis kit (Stratagene). GFP‐BAX and GFP‐BAX‐1‐2/L‐6 plasmids were kindly provided by N. Brady and F. Edlich, respectively. Finally, we thank Dr. Frank Essmann and Prof. Klaus Schulze‐Osthoff for providing the HCT116 BAX/BAK DKO cells.

### Purification and labeling of recombinant BCL2 family proteins

Mutant DNAs were generated by PCR-based mutagenesis using the Quickchange mutagenesis kit (Stratagene), or were purchased at GenTech. All constructs were verified by sequencing. BFL1∆C (lacking the C-terminal 24 residues) and its variants were expressed in *Escherichia coli* BL21 (DE3) using the pNIC28-Bsa4 vector (New England Biolabs). Cells were induced at OD of 1.5–2 with 1 mM IPTG, and grown overnight at 18°C. The harvested cells were lysed at 4°C with a Emulsiflex C5 homogenizer (Avestin) in 500 mM NaCl, 20 mM Imidazole, 50 mM Na_2_HPO_4_ pH 7.5, 1 mM TCEP, 10% glycerol, 1 mg/ml lysozyme, 2.5 µg/ml DNAse I, and complete EDTA-free protease inhibitor cocktail tablets (Roche). BFL1∆C proteins were purified from the supernatant by nickel-affinity and size-exclusion chromatography and stored in 100 mM KCl, 10 mM Hepes, pH 7.4, 1 mM EDTA (KHE buffer) supplemented with 1 mM TCEP and 10% w/v glycerol. BAX, cBID, BCLXL, BCLXLΔC, and their variants were expressed and purified as described [29]. All protein preparations were >95% pure as assessed by SDS-PAGE and Coomassie staining. In a typical protein labeling reaction, Alexa 488, Alexa 647 or NBD were incubated with BCL2 proteins at a molar ratio of 10:1, samples were incubated overnight at 4°C, and subsequently eluted over a PD-10 column equilibrated with KHE.

### Liposome preparation

To prepare GUV, approximately 5 µl of the lipid mixture stock in chloroform were spread on the platinum wires of the electroformation chamber. After solvent evaporation, the wires were immersed in 200 mM sucrose buffer, and electric pulses of 10 Hz were provided for 2 h, followed by 2-Hz pulses for 30 min. To prepare LUV, dry lipid films were suspended in KHE buffer, the resulting liposomes were subjected to 10 freeze/thaw cycles, and subsequently extruded 10 times through two polycarbonate membranes of 0.2-µm pore size (Nucleopore). Liposome lipid compositions were as follows (mol/mol): 0%CL, PC60/PE30/PI10/CL0; 25%CL, PC35/PE30/PI10/CL25; and 100%CL, pure CL. To prepare 25%CLox LUV, 25%CL LUV in EDTA-free KHE buffer were incubated with 20 μM CuCl_2_ at 37°C, and CL oxidation was checked by monitoring absorbance at 245 nm in a Uvikon 922 spectrophotometer (Kontron instruments).

### GUV confocal microscopy analyses

Protein recruitment to GUV, (S)FCCS, and GUV permeabilization experiments were all performed as described previously with small modifications [32]. Briefly, to assess protein recruitment to GUV, first fluorescently-labelled proteins were incubated with liposomes for 2 h at 37°C, followed by confocal fluorescence microscopy observation at 25°C, and image processing using the plug-in “Radial profile” of Image J. The amount of protein bound to the GUV was normalized to values obtained in solution. Protein concentrations were as follows: BAXr and BFL1ΔCg, 150 nM; cBID, 30 nM; and BFL1ΔC, BCLXL, and BCLXLΔC, 450 nM. FCCS measurements were performed at 25°C using a ConfoCor3 module with attenuated excitation light from argon ion (488 nm) and helium-neon lasers (633 nm). To obtain auto- and cross-correlation curves, raw fluorescence fluctuation data were fitted to a three-dimensional diffusion model with homemade software. Irregular curves resulting from instability and distortion were excluded from the analysis. To obtain surface concentrations of single colour particles (Cr and Cg) and two-colour particles (CCrg), auto- and cross-correlation functions were fitted with a nonlinear least-squares global fitting algorithm as described in [32]. FCCS results were also corrected for protein labelling degrees (BFL1ΔCg, 70%; BFL1ΔCg L21A, 75%; BFL1ΔCg R88D, 70%; BFL1ΔCr, 65%; BFL1ΔCr L21A, 70%; BFL1ΔCr R88D, 70%; BAXr 60%; and cBIDr 60%). Complex % values were calculated with respect to the amount of the green particles (CCrg X 100/Cg). Protein concentrations were 200 nM for assays in solution; 50 nM for assays with 25%CL GUV, and 20 nM for assays with 100%CL GUV. For GUV permeabilization assays, BCL2 family proteins were mixed in KHE buffer with FITC-4-kDa dextran, APC and GUV which had been doped with DiD. After 2 h incubation at room temperature, the percentage of internalization into the GUV of fluorescent probes was determined by confocal fluorescence microscopy and ImageJ software.

### Steady-state fluorescence spectroscopy

Fluorescence intensity and spectral analyses were done in an 8100 Aminco-Bowman luminescence spectrometer (Spectronic Instruments), in thermostatically controlled 4 × 4-mm quartz cuvettes, at 37°C. Assays of ANTS/DPX release and NBD-based fluorescence mapping were performed as previously described [29]. In all cases, the signal from background samples was subtracted from the sample fluorescence. To minimize vesicle light scattering, a 490 nm cut-off filter was placed in the emission light path. λmax values were determined from the first derivative of the smoothed spectra. Unless otherwise stated, BAX, BCLXL, and BFL1 concentrations were 300 nM, cBID concentration was 100 nM, and lipid concentration was 150 µM.

### TIRF experiments

To attain the single-molecule regime, LUV were incubated with 0.5 nM of BFL1∆Cg proteins for 1 h at room temperature and SLB were created on piranha-cleaned glass slides (Menzel). SLBs were immediately analysed using a modified Zeiss Axiovert 200 M epifluorescence microscope with a 488 laser equipped with a α Plan-Fluor 100×/1.46 oil objective (Zeiss), a Laser-TIRF 3 Imaging System (Zeiss) and a EM-CCD camera (iXon 897, Andor). Samples were illuminated for 35 ms with a delay time between frames of 25 ms and an intensity of 0.1 kW/cm^2^. The images acquired were used for the stoichiometry analysis based on the fluorescence intensity of the particles using an in-house algorithm implemented in Python (Python Software Foundation). Bright spots were automatically detected using an implementation of the difference of Gaussians method and thresholding. Selected particles were defined by a ROI of a defined pixel size (2 × 2) and fitted to two-dimensional (2D) Gaussians. Background subtraction was performed by defining a ROI around the particle´s ROI having a larger pixel size (3 × 3). Localized particles were discarded based on the distance and on the width of the 2D Gaussian, to avoid overlapping ROIs or multiple particles in the same ROI. This algorithm provided the brightness value for each spot. Stoichiometry counting was performed using the brightness analysis method as previously described [33]. Briefly, in the brightness analysis, theoretical brightness of higher oligomers is calculated based on the measured brightness of monomers. In order to measure the brightness of a monomer, individual monomeric BFL1∆Cg particles were obtained by spreading BFL1∆Cg particles directly on a cleaned glass coverslip and selected by photobleaching analysis after smoothing of the signal with a median filter. After obtaining a statistical relevant number of values, they were plotted as a histogram that was fitted to a Gaussian providing the mean intensity and SD of a single fluorophore (monomer). These values were used to calculate the theoretical mean and SD values for higher oligomers (N-mers). In Fig. 3d, the histograms represent the overall brightness distributions of individual particles (N-mers) of BFL1∆Cg (or its mutants). The overall brightness distribution was fitted as a sum of Gaussians imposing the theoretical mean and SD values, previously calculated for the different N-mers. The area under each curve was used to calculate the percentage of occurrence of each species, which was further corrected by taking into account that not all BFL1∆Cg molecules in a particle are labelled due to partial labelling (bar graphs in Fig. 3d). Correction for partial labelling efficiency was performed as previously described [33]. Only proteins having > 60% labelling efficiency were considered for experiments and data collection. The graphs with the distribution of species correspond to the average values obtained from three different experiments, and the error bars correspond to the averaged errors in the individual experiments, as they were larger than the S.D.

### Cellular analyses of intracellular protein distribution and nuclear morphology

HCT116 wt, BAX/BAK DKO or CL KO cells growing onto a coverslip were transiently transfected with jetPRIME for 16 h and treated with 1 µM rotenone/staurosporine for 0, 4, 8 or 16 h. Next, cells were incubated with 500 nM Mitotracker at 37°C for 30 min and washed twice with PBS before fixation with 3.8% paraformaldehyde. Coverslips were then immunoblotted with a primary anti cyt. c antibody (Abcam) and a secondary fluorescent anti-mouse 633 antibody (Life Technologies) and finally, the nuclei were dyed with Hoechst 33342 (Invitrogen). Samples were visualized in a Nikon TE2000 U inverted confocal microscope equipped with a D-eclipse C1si confocal spectral detector, using an X60, 1.45 numerical aperture, oil immersion objective. Images were processed by FiJi software. HCT116 CL KO cells [36], were a generous gift of Prof. Jean Claude Martinou.

### STED microscopy

HCT116 BAX/BAK DKO cells growing onto a coverslip were transfected with GFP-BFL1 and treated with 1 µM rotenone/staurosporine for 6 h as described above. Prior to fixation, cells were incubated with 1 μM Mitotracker at 37°C for 30 min. Next, samples were immunostained for anti-GFP (Abcam) and a secondary fluorescent anti-rabbit Alexa 488 (Life technologies). Images were acquired using an inverted confocal microscope Leica TCS g-STED CW SP8, equipped with a white light laser, high-sensitive hybrid detectors and 592 nm depletion laser for super-resolution (Gated STED CW) with 100x oil objective, at the Achucarro Basque Centre for Neuroscience- Imaging Facility (Leioa, Spain). Confocal microscopy and gSTED images were processed only for contrast stretching, and plot profiles along the GFP-BFL1 clusters for the gSTED-recorded images were obtained by Fiji. Plot profiles were fitted to a Gaussian function and cluster size estimated at Full Width at Half Maximum (FWHM) values.

## Supplementary information

Supplementary Material
