# Supplementary material for: BFL1 modulates apoptosis at the membrane level through a bifunctional and multimodal mechanism showing key differences with BCLXL
Source: Cell Death Differ. 2018 Dec 18;26(10):1880–94. doi: 10.1038/s41418-018-0258-5 (PMC6748131; doi:10.1038/s41418-018-0258-5)
Supplement: Supplementary file 1 — Supplementary Material [file 41418_2018_258_MOESM1_ESM.pdf]

Supplementary MATERIALS AND METHODS.

Cyt c release assay. BAX/BAK DKO mouse embryonic fibroblasts (MEFs) were harvested by scrapping, and homogenized with a glass-Teflon Potter-Elvehjem homogenizer in mitochondrial isolation buffer (210 mM mannitol, 70 mM sucrose, 10 mM Hepes (pH 7.5), 1 mM EDTA, and protease inhibitors). After removing heavy membrane fractions by two consecutive centrifugations at 700 *g* for 10 min at 4 °C, mitochondria-enriched fractions were pelleted by centrifuging the resultant supernatant at 14000 *g* for 10 min at 4 °C. Mitochondria (50 µg total protein) were incubated with recombinant BAX (100 nM), cBID (50 nM) and BFL1ΔC and its mutants in release buffer (125 mM KCl, 5 mM KH_2PO_4, 2 mM $MgCl_2$, 1 mM DTT, and 10 mM HEPES-KOH, pH 7,4), for 30 min at 30 °C. Samples were then centrifuged at 14000 *g* for 10 min, and supernatant and pellet fractions were subjected to SDS-PAGE and immunoblotting analysis using anti-cyt *c* 7H8.2C-12 (BD-Biosciences, San Jose, CA, USA). Immunoblotting using anti-Tom20 (Santa Cruz) was performed for mitochondrial localization determination and as loading control.

Protein structural validation. *Circular dichroism*. The CD spectra were collected using the following parameters a 1-nm step size over 260 to 200 nm wavelength range, speed 50 nm/min; response 1 s; bandwidth 2 nm in a spectropolarimeter Jasco J-810 (Jasco Spectroscopic Co. Ltd., Hachioji City, Japan) equipped with a JASCO PTC-423S temperature control unit. Each measurement was the average of 20 scans, which was background- subtracted. All samples were allowed to equilibrate for 10 min prior to CD analysis. Secondary structure content was estimated from the far-UV spectra using CDPro software. To assess the thermal stability of the BCL2 proteins studied, CD data was collected at 222 nm wavelength every 1°C from 25°C to 90°C. The midpoint of the melting transition was estimated using the first derivative converter available in the software of the CD instrument with a window of nine data points and a 2nd degree polynomial smoothing. Protein concentration in all cases was 3 µM in PBS buffer. *Tryptophan fluorescence spectra.* Fluorescence intensity and spectral analyses were done in an 8100 Aminco-Bowman luminescence spectrometer (Spectronic Instruments, Rochester, NY), in thermostatically controlled 4x4-mm quartz cuvettes, at 25°C. Trp spectra were recorded between 305 nm and 405 nm at a scan rate of 1 nm/s, using an excitation wavelength of 295 nm (slits 4 nm). Protein concentration in all cases was 1 µM in KHE buffer.

Oligomerization assay by size exclusion chromatography. BCL2 proteins (5µM) were incubated in KHE for 1h at 37°C with or without 100%CL LUV (500µM lipid), followed by addition of 3% CHAPS (w/v) and further incubation for 30 min at room temperature. Samples were loaded in a Superdex 75 10/300 column pre-equilibrated in KHE buffer supplemented with 3% CHAPS. Protein standards were: ribonuclease A (13.7 kDa), Carbonic anhydrase (29 kDa), Conalbumin (75 kDa) and Aldolase (158 kDa).

Supplementary FIGURE LEGENDS

Fig. S1. **BFL1 and BAX are in a codependent dynamic equilibrium between cytosol and the OMM.** (**a**) Pictorial scheme of experimental design: (a) Before bleaching, GFP-BAX was localized in mitochondria and cytosol; (b) FLIP bleaches cytosolic GFP-BAX; (c) bleached GFP-BAX molecules translocate to the mitochondria while fluorescent GFP-BAX retrotranslocates into the cytoplasm; (d) After continued FLIP, all GFP-BAX molecules are bleached. (**b**) GFP-BAX fluorescence monitorization in a typical FLIP experiment. Region marked as a white square was bleached 20 times at 488nm. GFP-BAX fluorescence variations on the mitochondria are detected in two areas (red circle and green circle, respectively), and changes in the cytosolic fluorescence (blue circle). Black and yellow circles indicate adjacent cells fluorescence and intercell fluorescence respectively, which serves as a control for cell-specific bleaching. (**c**) Cytosolic GFP-BAX fluorescence was monitored during FLIP assay in the absence (green line) or presence of BFL1 (blue line). Control represents GFP-BAX fluorescence in a neighbor cell (black line). (**d**) Mitochondrial GFP-BFL1 fluorescence was monitored during FLIP assay. Left, representative images showing time-dependent FLIP of GFP-BFL1 in the absence (top) and presence (bottom) of overexpressed BAX. Right, average FLIP kinetics for multiple cells expressing only GFP-BFL1 (green line) and in the presence of BAX (blue line). Control represents GFP-BFL1 fluorescence in a neighbor cell (black line). (**e**) Mitochondrial GFP-BAX 1-2/L-6 fluorescence was monitored during FLIP assay. Left**,** representative images showing time-dependent FLIP of GFP-BAX 1-2/L-6 in the absence (top) and presence (bottom) of overexpressed BFL1. Right, average FLIP kinetics for multiple cells expressing only GFP-BAX 1-2/L-6 (red line) and in the presence of BFL1 (purple line). Control represents GFP-BAX 1-2/L-6 fluorescence in a neighbor cell (black line). (**c, d, e**) Average FLIP kinetics, n>20 ROI for each condition of 3 independent experiments. *Error bars*, S.D. (**c, d**) Arrows denote mitochondria. (**b, d**, **e**) Scale bars, 10µm. ***, p= <0.001.

Fig. S2. BFL1ΔCg, BAXr, and cBIDr maintain structural and functional properties of native unlabeled counterparts. (**a**) Three-dimensional structures of BFL1ΔC (PDB code 2VM6), BAX (PDB code 1F16), and BID (PDB code 1DDB) displaying as colored spheres the monocysteine residue where the alexa-fluorophore is conjugated to generate BFL1ΔCg, BAXr, and cBIDr variants used in this study. (**b**) Percentage of ANTS/DPX permeabilized 25%CL LUV in the presence or absence of the indicated BCL2 proteins at room temperature. Mean values ± S.D. (*error bars*) correspond to 3 independent experiments. (**c**) Effect of BCL2 proteins on the release of cytochrome *c* from mitochondria isolated of MEF BAX/BAK DKO cells. (**d**) as in (**b**) but at 43°C. (**b, c, d**) Protein concentration: cBID($_{r}$)=150nM, BAX($_{r}$)=150nM and BFL1ΔC($_{g}$)=450nM). (**e**) Representative Trp fluorescence spectra, thermal denaturation assay and circular dichroism spectra of BFL1ΔC WT and its canonical and non-canonical mutants**.** (**f**) Assessment of cyt c release inhibition by BFL1ΔC and its mutants in isolated mitochondria from MEF BAX/BAK DKO cells. (**g**) Representative emission spectra of NBD-BAX variants in solution (dotted lines), with 25%CL LUV (dashed lines) or with LUV and cBID (continuous lines). In red those residues located in the canonical ligand (T56 and C62) and in cyan the non-canonical surface (M74, D84 R89, C126 and I133).

Fig. S3. (**a**) Controls for FCCS and SFCCS. In FCCS, we measured the %CC between BFL1ΔCg and Alexa647 and BFL1ΔCr and Alexa488 and SFCCS we analyzed the %CC between BFL1ΔCg and DID, BFL1r and DIO stablishing the background levels in 100%CL GUV. For the dynamics in the homocomplex formation we analyzed the %CC between BFL1ΔCg (20nM):BFL1ΔCr (20nM): BFL1ΔC (60nM) in 100%CL GUV. Box chart and raw data representation of complex formation. Plot details were described in Fig. 1b. (**b, c**) cBIDr promotes BFL1ΔCg binding to the membrane.

BFL1ΔCg and cBIDr were incubated with GUV containing different amounts of CL followed by analysis of protein localization by confocal fluorescence microscopy. Vesicle composition and box chart and raw data representation as described in Fig.1b.

Fig. S4. Quaternary structure study of BFL1ΔC, BCLXL, BAX and its variant. Representative curves of BCL2 proteins oligomerization state. Proteins were incubated in the absence (dashed lines) or in the presence of 100%CL LUVs (solid lines) followed by 3% CHAPS addition, and Superdex-75 chromatography. Analyzed proteins were: (**a**) wt proteins; BFL1ΔC (black lines), BCLXL (grey lines) and BAX (dark blue lines) and (**b**) canonical mutants, BFL1ΔC R88D (red lines) and BAX R109D (dark purple lines). (**a, b**) (n=3). (**c**) Membrane permeabilization elicited by BFL1ΔC L21A, BFL1ΔC R88D and BAX R109D in apoptotic-like LUV. Protein concentration: Dotted bars, 60nM; Dashed bars, 120nM; filled bars, 240nM. n≥ 3 technical replicates. Error bars, SE. (**d**) Dose-dependence of extents of cBID-activated BAX vesicular contents release inhibition elicited by BFL1ΔC (black line), BFL1ΔC K101EK102E (orange line) and BFL1ΔC K146EK147E (green line) in 25%CL LUV. (n = 3) Error bars, S.D.

FIG. S5. Effects of rotenone in GFPBFL1, GFPBFL1 KKEE, GFP and Mito-GFP. (**a**) Representative images of GFPBFL1 and GFPBFL1 K101E/K102E cellular distribution (in green), cyt c localization (in blue) and mitochondria (in red) in the presence or absence of rotenone (1μM, 4h) in HCT116 cells. (**b**) Representative images of GFP and Mito-GFP cellular distribution (in green) and cyt c localization (in blue) in the presence/absence of rotenone (1μM, 4h) in HCT116 cells. (**a, b**) Scale bars, 10μm**.** (**c**) Time-dependence of cluster formation (left) and cyt c release (right) in GFP/mito-GFP transfected HCT116 cells treated or untreated with rotenone (1 μM). At least 50 cells were analyzed per condition and experiment, and all the experiments were performed at least three times. Data are expressed as mean ± SD. (**d**) Analysis of the expression levels of ectopically expressed GFPBFL1, GFPBFL1 L21A, GFPBFL1 R88D and GFPBFL1 K101E/K102E in HCT116 cells. Loading control was determined by anti-Tom20 antibody and Ponceau staining.

FIG. S6. (**a**) GFPBAX, GFPBCLXL and GFPBFL1 expression levels in HCT116 wt and BAX/BAK DKO cells by western blotting**.** Loading control was determined by anti-Tom20 antibody. (**b**) Percentage of transfected HCT116 BAX/BAK DKO cells with GFPBFL1 or GFP, presenting clusters, cyt c release, or apoptotic nuclei in the presence or absence of rotenone treatment (1μM, 6h). Data represent mean ± SD from three independent experiments (n>50 on each repetition). (**c**) Percentage of GFPBFL1, GFPBFL1 L21A AND GFPBFL1 R88D transfected HCT116 BAX/BAK DKO cluster presenting cells with cyt c release after staurosporine treatment (1μM, 6h). Data represent mean ± SD from three independent experiments (n>50 on each repetition). **, *p*= <0.025. *Error bars*, S.D.

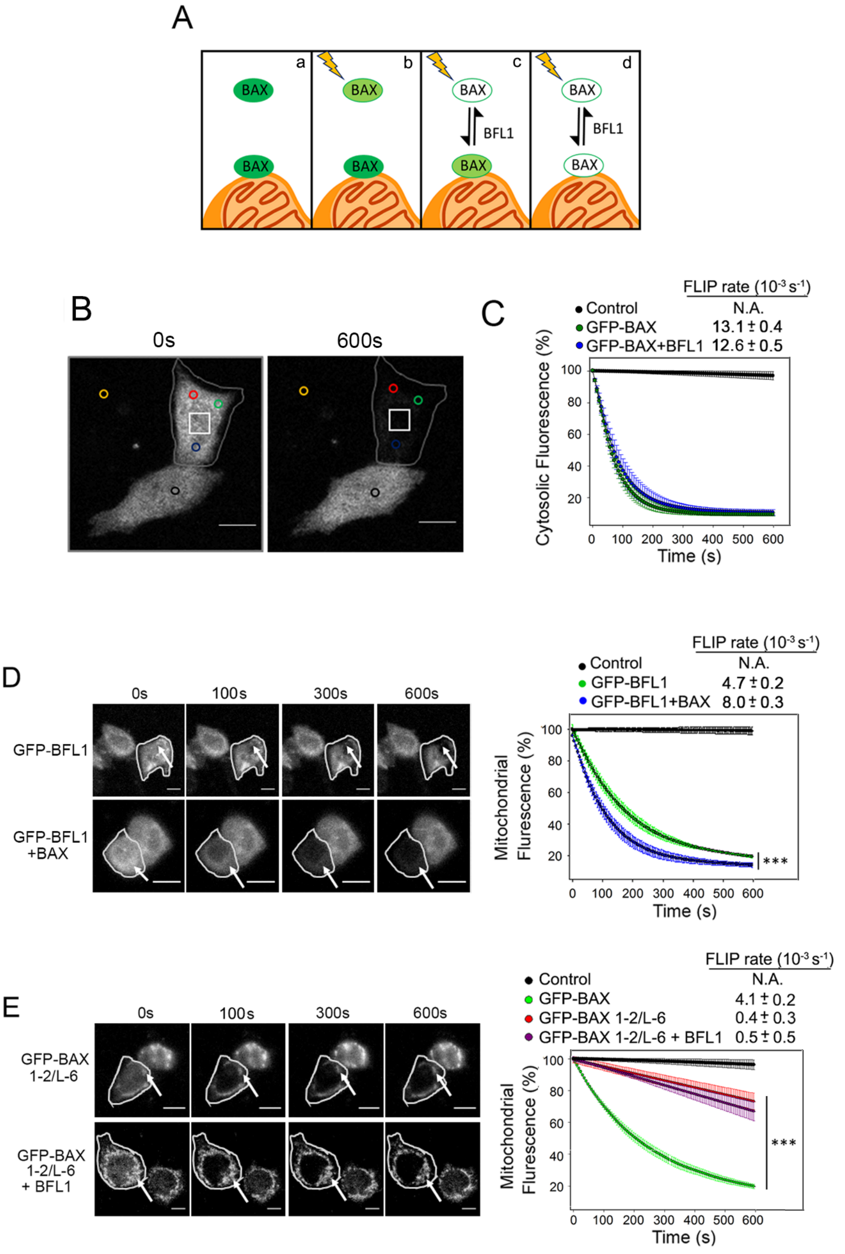

A
a
b
c
d
BAX
BFL1
B
0s
600s
C
FLIP rate (10-3 s-1)
Control N.A.
GFP-BAX 13.1 ± 0.4
GFP-BAX+BFL1 12.6 ± 0.5
Cytosolic Fluorescence (%)
Time (s)
D
0s
100s
300s
600s
GFP-BFL1
GFP-BFL1 +BAX
FLIP rate (10-3 s-1)
Control N.A.
GFP-BFL1 4.7 ± 0.2
GFP-BFL1+BAX 8.0 ± 0.3
Mitochondrial Fluorescence (%)
***
Time (s)
E
0s
100s
300s
600s
GFP-BAX 1-2/L-6
GFP-BAX 1-2/L-6 + BFL1
FLIP rate (10-3 s-1)
Control N.A.
GFP-BAX 4.1 ± 0.2
GFP-BAX 1-2/L-6 0.4 ± 0.3
GFP-BAX 1-2/L-6 + BFL1 0.5 ± 0.5
Mitochondrial Flurescence (%)
***
Time (s)

FIGURE S1

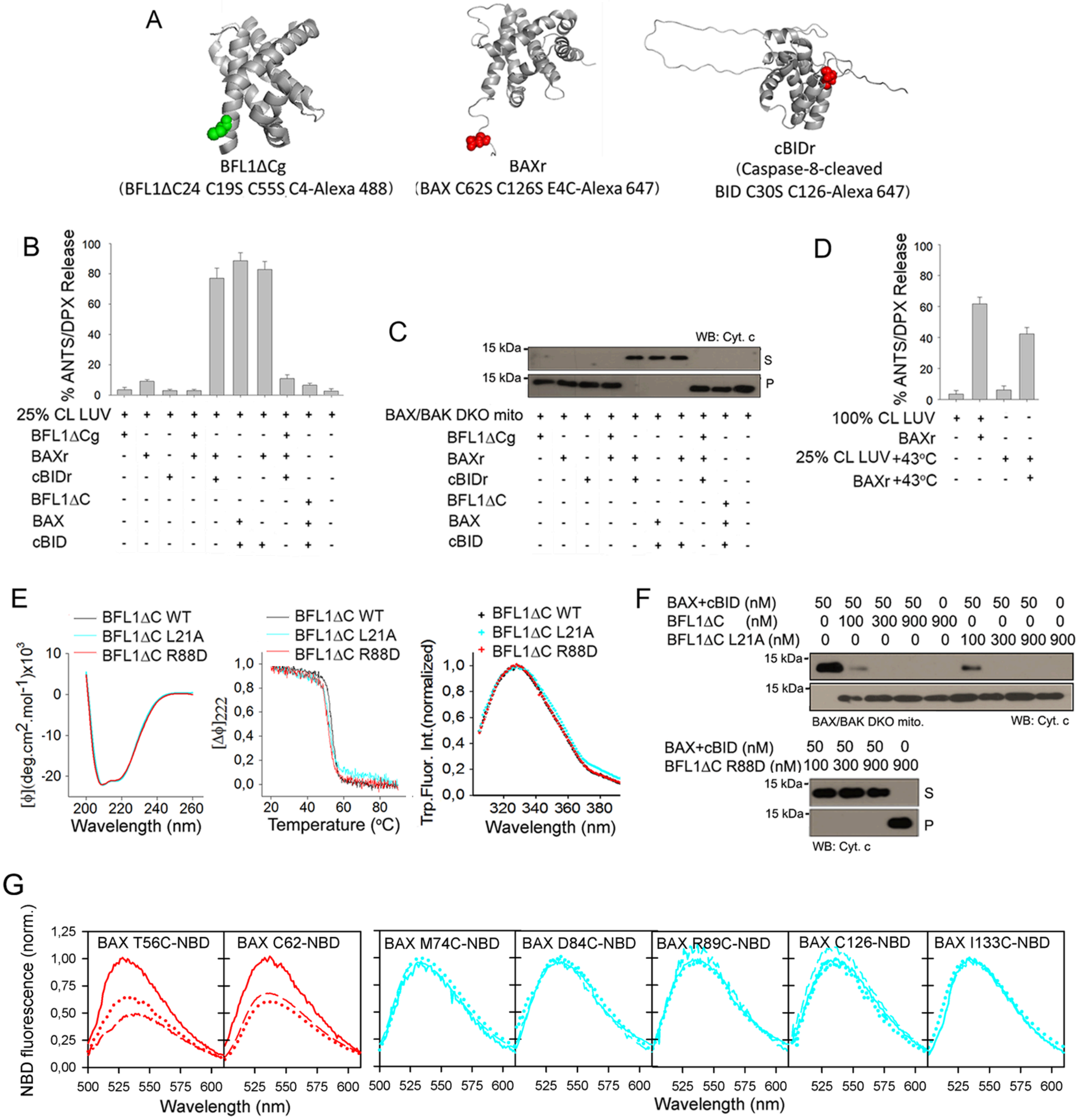
A
BFL1ΔCg
(BFL1ΔC24 C19S C55S C4-Alexa 488)
BAXr
(BAX C62S C126S E4C-Alexa 647)
cBIDr
(Caspase-8-cleaved
BID C30S C126-Alexa 647)
B
% ANTS/DPX Release
25% CL LUV
BFL1ΔCg
BAXr
cBIDr
BFL1ΔC
BAX
cBID
C
WB: Cyt. c
15 kDa
S
P
BAX/BAK DKO mito
BFL1ΔCg
BAXr
cBIDr
BFL1ΔC
BAX
cBID
D
% ANTS/DPX Release
100% CL LUV
BAXr
25% CL LUV +43ºC
BAXr +43ºC
E
BFL1ΔC WT
BFL1ΔC L21A
BFL1ΔC R88D
[ϕ](deg.cm2.mol-1)x103
Wavelength (nm)
[Δϕ]222
Temperature (ºC)
Trp.Fluor. Int.(normalized)
Wavelength (nm)
F
BAX+cBID (nM) 50 50 50 50 0 50 50 50 0
BFL1ΔC (nM) 0 100 300 900 900 0 0 0 0
BFL1ΔC L21A (nM) 0 0 0 0 0 100 300 900 900
15 kDa
BAX/BAK DKO mito.
WB: Cyt. c
BAX+cBID (nM) 50 50 50 0
BFL1ΔC R88D (nM) 100 300 900 900
15 kDa
S
P
WB: Cyt. c
G
NBD fluorescence (norm.)
BAX T56C-NBD
BAX C62-NBD
BAX M74C-NBD
BAX D84C-NBD
BAX R89C-NBD
BAX C126-NBD
BAX I133C-NBD
Wavelength (nm)
Wavelength (nm)

FIGURE S2

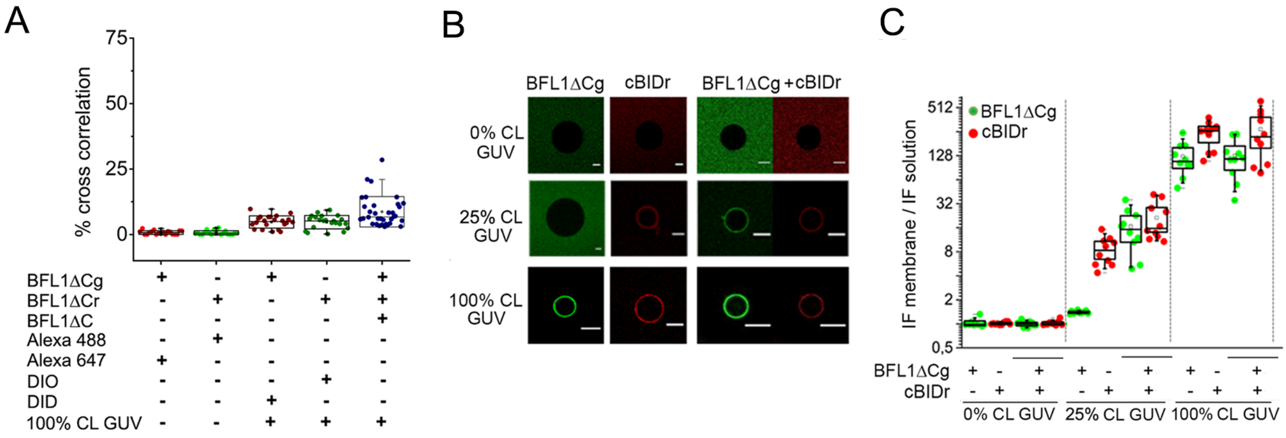
A
% cross correlation
75
50
25
0
BFL1ΔCg
BFL1ΔCr
BFL1ΔC
Alexa 488
Alexa 647
DIO
DID
100% CL GUV
B
BFL1ΔCg
cBIDr
BFL1ΔCg +cBIDr
0% CL GUV
25% CL GUV
100% CL GUV
C
BFL1ΔCg
cBIDr
IF membrane / IF solution
512
128
32
8
2
0,5
0% CL GUV
25% CL GUV
100% CL GUV

FIGURE S3

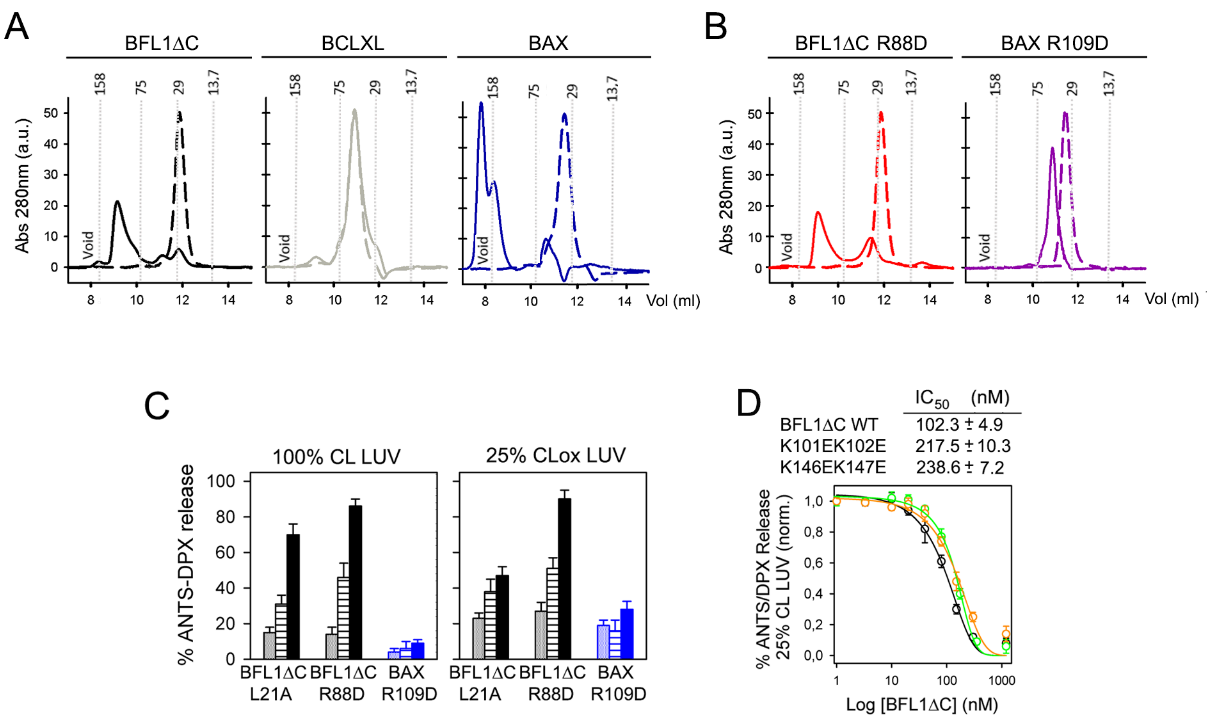

A
BFL1ΔC
BCLXL
BAX
B
BFL1ΔC R88D
BAX R109D
Abs 280nm (a.u.)
Void
158
75
29
13,7
Vol (ml)
C
100% CL LUV
25% CLox LUV
% ANTS-DPX release
BFL1ΔC L21A
BFL1ΔC R88D
BAX R109D
D
IC50 (nM)
BFL1ΔC WT 102.3 ± 4.9
K101EK102E 217.5 ± 10.3
K146EK147E 238.6 ± 7.2
% ANTS/DPX Release 25% CL LUV (norm.)
Log [BFL1ΔC] (nM)

FIGURE S4

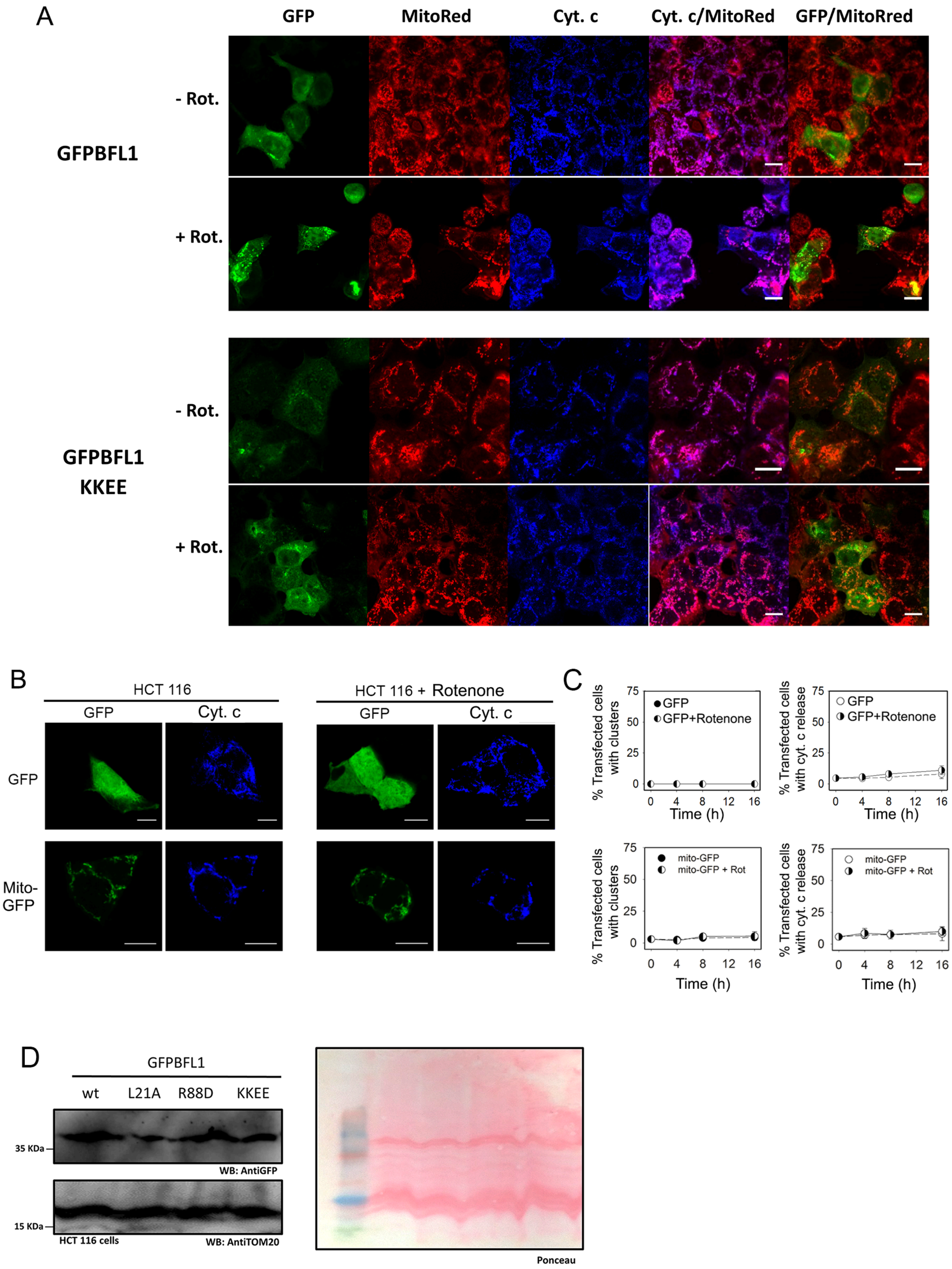
A
GFP
MitoRed
Cyt. c
Cyt. c/MitoRed
GFP/MitoRred
- Rot.
+ Rot.
GFPBFL1
GFPBFL1 KKEE
B
HCT 116
HCT 116 + Rotenone
GFP
Cyt. c
Mito-GFP
C
% Transfected cells with clusters
% Transfected cells with cyt. c release
GFP
GFP+Rotenone
mito-GFP
mito-GFP + Rot
Time (h)
D
GFPBFL1
wt
L21A
R88D
KKEE
35 KDa
WB: AntiGFP
15 KDa
HCT 116 cells
WB: AntiTOM20
Ponceau

FIGURE S5

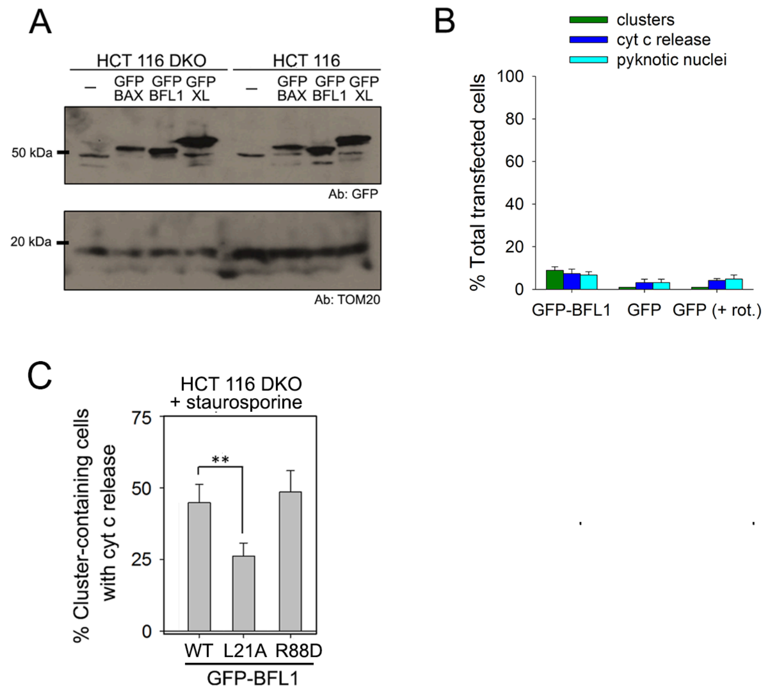
A
HCT 116 DKO
HCT 116
GFP BAX
GFP BFL1
GFP XL
50 kDa
Ab: GFP
20 kDa
Ab: TOM20
B
clusters
cyt c release
pyknotic nuclei
% Total transfected cells
100
80
60
40
20
0
GFP-BFL1
GFP
GFP (+ rot.)
C
HCT 116 DKO
+ staurosporine
% Cluster-containing cells with cyt c release
75
50
25
0
**
WT
L21A
R88D
GFP-BFL1

FIGURE S6
